# Definition of reproductive structures in *Eucalyptus* for phenological data collection

**DOI:** 10.1007/s00484-024-02820-4

**Published:** 2024-11-14

**Authors:** Claudia Helena Giraldo Escobar, Marie R. Keatley, Sabine Kasel, Julian Di Stefano, Craig R. Nitschke

**Affiliations:** 1 School of Agriculture, Food and Ecosystem Sciences, Faculty of Science, The University of Melbourne, Burnley, Victoria 3121 Australia; 2 School of Agriculture, Food and Ecosystem Sciences, Faculty of Science, The University of Melbourne, Creswick, Victoria 3363 Australia; 3 Applied Chemistry and Environmental Science, RMIT University, Melbourne, Victoria 3000 Australia

**Keywords:** Plant reproductive phenology, Herbarium records, *Eucalyptus baxteri*, Fruit formation, Floral bud development

## Abstract

In an era where global climate change is shifting plant phenology, global meta-analyses of multiple species are required more than ever. Common language or references for enhanced data compatibility are key for such analyses. Although the Plant Phenology Ontology (PPO) addresses this challenge, it does not capture several relevant reproductive structures that are critical in species with long reproductive cycles, like many *Eucalyptus* species. We reviewed the terminology and concepts that describe reproductive structures in eucalypts and compared them with the existing classes of the PPO to explore the PPO’s potential for harmonizing disparate eucalypt datasets. We identified incongruencies within and between eucalypt terminology and the PPO. We tested the sensitivity of the PPO for capturing key eucalypt phenological structures and found it sensitive to classification of certain structures. To address these limitations, we developed the *Eucalyptus* Phenology Ontology (EPO), a new ontology that builds on the PPO and captures key reproductive structures using a more refined classification. The EPO integrates the relationships between reproductive structures, phenological stages, and phenological traits. The vocabulary is species-neutral so it can be applied to other taxa but specifies the synonyms and descriptions required to capture the complexity of eucalypt phenology.

**Supplementary Information:**

The online version contains supplementary material available at 10.1007/s00484-024-02820-4.

## Introduction

Plant phenology is the science of the timing of recurrent cycles such as leafing out, growth, flowering and fruiting, and their association with climatic signals (Lieth 1974). Understanding plant phenology allows us to quantify the interactions between plants and fauna (e.g., Giles et al. 2016; Morellato-Cerdeira et al. 2016), climate (e.g., Keatley et al. 2002; Stephens et al. 2022), and human activities (e.g., Arundel et al. 2016; Nagai et al. 2019; van Vliet 2010). For wild plants, long-term phenological data are often scarce, limiting the ability to study these critical interactions. Aggregating diverse data sources such as herbarium collections, satellite imagery, monitoring data, and observations, is key to addressing this challenge, and developing a common language for describing the phenological traits is necessary to allow effective data harmonization (Brenskelle et al. 2019; Stucky et al. 2018).

Unlike most of the northern temperate angiosperm tree species, eucalypt reproductive phenology is characterized by long reproductive cycles that overlap along a developing branchlet (see Fig. S1 in Online Resource 1). This is especially the case in southeastern Australian eucalypts of the subgenus *Eucalyptus*. For example, the length of the reproductive cycle of *Eucalyptus regnans* (Ashton 1975), *E. baxteri* (Andersen 1989; Koch 2003), *E. sieberi* (Bassett 2002) occurs over five years from floral bud initiation to fruit fall. The floral bud development stage: from bud initiation to anthesis, can last two years or more in these species. Apart from the biological and phenological effects a long cycle can have, this length has influenced the language used to describe the different stages and levels of classification of the reproductive structures in eucalypts. The longer the cycle, the more details there are to observe. Commonly, the literature on eucalypt reproductive patterns defines two to four stages of floral buds (e.g., Bassett 2002; Gill 1997; Salter 2023), and two to four stages of fruits (Andersen 1989; Bassett 2002; Davis 1969). The terms used, and their meanings, have differed from study to study, which may obstruct efforts to compare between studies and lead to ambiguity (Gortner et al. 1967).

Considerable effort has been devoted to standardizing vocabulary and promoting interoperability with other vocabulary systems (Bruns and van Vliet 2003; Cox et al. 2021; Falster et al. 2021; Yost et al. 2018). An example of this was the creation and promotion of the numeric BBCH scale to describe phenophases (Meier et al. 2009). Although the BBCH scale harmonized the vocabulary its applicability is limited because: (1) it is conceptualized on northern temperate models of phenology and its applicability to non-deciduous plants is not straightforward, especially for those with long overlapping cycles like eucalypts, (2) it needs to be adapted on a species-by-species basis, hindering the interoperability between different taxa; and, (3) the phenophase descriptions produce qualitative information that confound data modeling, reuse, and harmonization. Such barriers are overcome by quantifying phenological traits (Langvall & Ottosson Löfvenius 2021; Stucky et al. 2018).

The Plant Phenology Ontology (PPO, Stucky et al. 2018) was developed to facilitate the description of phenological traits on samples and facilitate the aggregation of diverse phenological data from multiple sources (Stucky et al. 2018). The PPO is part of the Open Biological and Biomedical Ontologies (OBO, https://obofoundry.org/), which sets out the principles required for development of ontologies and constitutes a central location for interoperable ontologies (Smith et al. 2007). The PPO built upon the Plant Ontology (PO) framework (Walls et al. 2019), which focused on anatomy, morphology, and development stages; both, in turn, are connected to the Gene Ontology (GO) framework, which defines biological processes (du Plessis et al. 2011).

The focus of PPO on observable traits, like “20 open flowers present on the tree” rather than conceptual processes like “the tree is in the flowering phenophase”, facilitates quantitative data collection, use, reuse, and modeling (Stucky et al. 2018). The PPO explicitly connects a ‘plant phenological trait’ with a ‘continuant entity’ (‘reproductive structure’) and an ‘occurrent entity’ (‘plant structure development stage’). For example, ‘open flower presence’ is a trait that is a ‘quality of’ a ‘whole plant’ that has at least one ‘open flower’, and an ‘open flower’ is a ‘flower’ in the ‘open flower stage’. The definition of the reproductive structures (RS) is carried to every level of the ontology.

This paper is divided into two sections. The first aims to present a critical review of both (1) the terminology that describes eucalypt RS, and (2) the PO and PPO terminology and their applicability to eucalypts. The review forms the basis for a new *Eucalyptus* Phenology Ontology (EPO). The new ontology provides species-neutral classes that map to the PO and PPO. Developing this framework is important for harmonization of historical data collected using different approaches. Importantly, this ontology provides an approach for standardizing future collection of phenological data in eucalypts in a consistent manner with the PPO model and standards. The second section aims to analyze the ecological relationships between the RS classes, determine the relevance of classifying the eucalypt RS in a narrower manner, and defining key phenophases in eucalypts.

## Reproductive structures: terms in eucalypt literature in contrast with the PO and PPO ontologies

Unlike the PPO, which classifies RS into flowers and fruits, in eucalypts RS are divided into buds, flowers, and fruits. The long reproductive cycles of eucalypts make their intermediate stages important; therefore, diverse names, phrases and definitions have been created to capture these stages. One major feature of eucalypt terminology is the use of the word ‘bud’ to indicate a ‘reproductive bud’ (*sensu* PO) or an ‘unopened floral structure’ (*sensu* PPO). In contrast with phenological studies in other taxa, in eucalypt literature, ‘bud’ rarely represents a ‘vegetative bud’ (CANBR 2020). As this article is about reproductive phenology, the term ‘bud’ is expressed in a manner that is consistent with its use in eucalypts. Also, the RS, from flower buds to fruits, are aggregated into “umbels”, which emerge from an inflorescence bud. The words ‘umbel’ and ‘cluster’ are used to indicate both ‘inflorescence’ and ‘infructescence’. Finally, because the eucalypt fruit is a ‘circumscissile capsule fruit’ (as defined in the PO) and is commonly referred to as a ‘capsule’ (e.g., Bassett 2002; Dooley et al. 2010; Koch 2003), we use capsule as a synonym of fruit. This section aims to illustrate the different terminology and the different way terms are applied to eucalypt RS and how they compare with global standards and concepts.

### Inflorescence buds

**Fig. 1:**
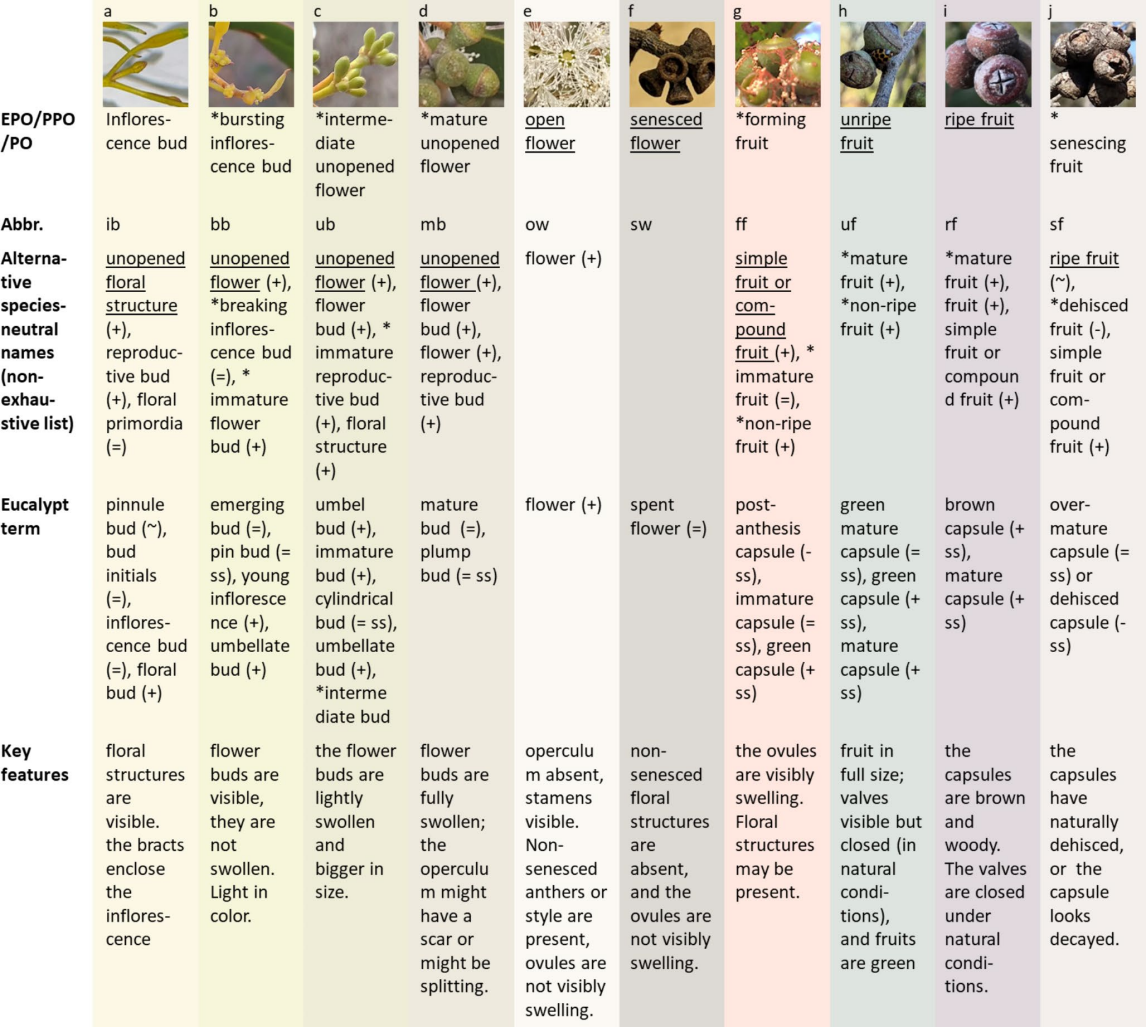
Selection of the ten reproductive structures included in the Eucalyptus Phenology Ontology (EPO); sorted in ascending order of development (left to right). From top to bottom: Pictures of Eucalyptus baxteri s.l. reproductive structures; selected name; associated abbreviation; alternative synonymous names; names commonly used to refer to eucalypt reproductive structures; and description of the reproductive and key characteristics for classifying them in eucalypts. Synonym type: “=”: exact synonym, “-”: narrow synonym, “+” broad synonym, “~” related, “ss” species- or taxon-specific. “*” indicates the terms that are not part of the PO nor PPO. Underlined: the narrowest or most detailed term that the PPO mapping offers to the structure. Pictures: Claudia Helena Giraldo Escobar

Before the eucalypt flower buds are visible and arranged in an umbellate inflorescence, they are enclosed by bracts. This RS is a bud that develops into an inflorescence, referred to as an ‘inflorescence bud’ in the PO (Fig. 1a). The term ‘inflorescence bud’, as defined earlier, has been used in eucalypts by Ashton (1975), Bassett (2002), Davis (1969) and Rawal et al. (2015a). Semple and Koen (2020) used the term ‘bud initials’ to define these structures, while Andersen (1989), Jones et al. (2011), and Moncur and Boland (1989) referred to this structure as a ‘floral bud’. The term ‘floral bud’ should be used with care, as it can refer to a flower, an inflorescence, or areproductive bud. Another ambiguous term is ‘young inflorescence.’ Boland et al. (1980) used it to mean ‘inflorescence bud’, while Potts and Gore (1995) used it to define the flower buds revealed when the bracts of the inflorescence bud open. The term ‘bracteate inflorescence’ (Moncur and Boland 1989), however, is species-specific, as eucalypts have bracts that protect the inflorescence. Therefore, it should only be used in the ontology as a synonym. Inflorescence buds could be associated with the PPO classes: ‘inflorescence bud’ and ‘unopened flower head’. These two classes are unrelated, highlighting that even within ontologies, terms can be puzzling. To avoid confusion, the meanings and relationships between the terms must be clear. Here we use ‘inflorescence bud’ for this type of structure.

### Flower buds

The flower buds are an essential part of what defines a eucalypt taxonomically. Etymologically, *Eucalyptus* means “well covered” in reference to the operculum, a cap-like structure that covers the anthers and other floral structures (Williams and Brooker 1997), from which dehiscence marks the beginning of anthesis (e.g., Ellis and Sedgley 1992; Griffin 1980; Jones et al. 2011). Due to the longevity of the eucalypt flower buds, from one to three years in some species (Andersen 1989; Ashton 1975; Bassett 2002), different names have been given to the structure according to the stage of development. Primarily, if not termed ‘flower bud’, most authors use the term ‘umbel bud’ (Rawal et al. 2015a) or ‘umbellate bud’ (Ashton 1975; Bassett 2002), to indicate that they belong to an umbel-like inflorescence. In some cases, they are referred to as an ‘unopened bud’ (Moncur and Boland 1989) or a ‘closed bud’ (Salter 2023). In reference to ‘open flower’, it is common to find the verb ‘flower’ associated with the noun ‘bud’ as in: “buds flowering” (field data sheets, pers. obs.), “buds flower” (Ashton 1975), or as described by Salter (2023), “opening bud”. This terminology reflects the critical importance that flower buds have in eucalypt phenological studies. This bud-centered terminology contrasts with the flower-centered terminology of the PPO. The PPO refers to these ‘flower buds’ as ‘unopened flowers’ because the term ‘flower’ applies to the RS from the ‘meristem transition stage’ to ‘flower senescence’ (as defined in the PO).

Eucalypt flower buds are typically subclassified into ‘mature bud’ (Fig. 1d) and ‘immature bud’ (Gill 1997; Spencer et al. 2020), but Semple and Koen (2020) subclassify buds into three stages: pin, cylindrical and plump. The term ‘mature bud’ or ‘mature flower bud’ is equivalent to a ‘plump bud’ (*sensu* Semple and Koen 2020), ‘large bud’ (*sensu* Davis 1969), “buds that are ready to open” (Salter 2023) or “umbellate bud just prior to anthesis” (Bassett 2002). Despite the many ways in which authors have referred to this structure, ‘mature flower bud’ (or ‘mature unopened flower’ in the PPO sense) is an unambiguous term; however, this is not the case for ‘immature bud’.

The term ‘immature bud’ can refer to all development stages from ‘inflorescence bud’ to bud maturity, meaning “all the types of reproductive buds that are not mature” (see Gill 1997; Spencer et al. 2020) or it may refer to any stage of the flower buds. Here, we use the term ‘immature flower bud’ for a ‘flower bud’ that has not reached maturity or ‘immature floral bud’ to allow the inclusion of inflorescence buds. Additionally, ‘immature flower bud’ may further be divided into two subclasses, as follows. In addition to Semple and Koen’s (2020) classification, other definitions are: ‘young inflorescence’ (Potts and Gore 1995), ‘emerging bud’, ‘embryonic bud’ (Koch 2003), or “umbellate buds just initiated” (Bassett 2002). For this structure, we propose the term ‘bursting inflorescence bud’ (Fig. 1b), which relates to the ‘bud burst stage’ that is included in the PO. Having separated the bursting inflorescence buds, the remaining structure is the intermediate flower bud structure that occurs between the ‘bursting inflorescence bud’ and the ‘mature flower bud’. This structure was named as ‘cylindrical bud’ by Semple and Koen (2020), but it is commonly called an ‘umbel bud’. As these two names are taxon-specific we propose the term ‘intermediate flower bud’ (Fig. 1c).

### Structures in anthesis (open flowers) and post-anthesis stages

According to the PO, the flowering stage initiates when anthers dehisce. Evaluating anther dehiscence to define anthesis initiation in eucalypts is impractical because it is the operculum dehiscence that is consistently used as an indicator of anthesis (Fig. 1e; e.g., Griffin 1980; Jones et al. 2011; Potts and Gore 1995). Additionally, anthers can dehisce before the operculum is shed (Moncur and Boland 1989). The completion of the ‘open flower stage’ is also vaguely defined for eucalypts.

Different definitions have been used to determine the cessation of the flowering stage. Style abscission has been suggested as a reference point (Moncur and Boland 1989; Ellis and Sedgley 1992). Ashton (1975) pictorially suggests complete stamen abscission as an indication of the completion of flowering. Brooker and Kleinig (1990) indicated that flowering has ceased by the time the stamens begin to senesce. Work by Bassett (1995) found that style abscission occurred after stamen abscission in *E. globoidea*. Further, Polunina (1959 in Davis 1968 p32) suggested “appreciable enlargement of the ovary” as the cessation of flowering.

The ‘flowering stage’, according to the PO, ends with flower senescence or pollination; the latter marks the onset of the ‘fruit development stage’. Botanically, inflorescences/flowers and infructescences/fruits are very different entities. The former develop from a meristem into a reproductive shoot system, while the latter develop from the ovaries of the flowers. Nevertheless, this theory is not always applied in practice. For example, Dooley et al. (2010) considered a RS to be a flower if floral structures were still attached, even if the ovules were evidently swelling; while Bassett (2002) recognized these structures as “immature capsules” but classified them as flowers. It is common practice in eucalypt phenological monitoring to include these post-pollination structures as flowers (see Fig. S2 in Online Resource 1). Furthermore, PPO specialists have classified the *Prunus* sp. “early fruits” as ‘senesced flowers’ (Brenskelle et al. 2019). The post-pollination structures in eucalypts are also sometimes acknowledged as fruits, under the names of: ‘young green fruit’ (Davis 1969), ‘capsule post-anthesis’ (Spencer et al. 2020), ‘immature fruit’ (Andersen 1989), ‘immature post-flowering capsules’ (Gill 1997); and “spent flower or young capsule” (Salter 2023).

Considering the botanical definition of fruit in the PO and the acknowledgment given by some authors, we consider that a RS is a ‘fruit’ when the ovules are swelling, even if the RS has floral structures attached. The post-pollination stage is named the ‘fruit formation stage’ in the PO, but a respective RS has not been defined as is the case for the ‘fruit ripening stage’, termed ‘ripening fruit’. To keep a similar terminology, we chose to name ‘forming fruit’ to the ‘fruit’ that is in the ‘fruit formation stage’ (Fig. 1g, Fig. S2 Online Resource 1). The ‘fruit formation stage’ refers to the process of cellular enlargement until the fruit reaches its full size and commences the ‘fruit ripening stage’. This distinction is well defined in stages 7 and 8 of the BBCH scale (Meier et al. 2009), although the terms ‘formation’ and ‘development’ are used synonymously. In eucalypts, ‘forming fruit’ is distinguished from ‘ripening fruit’ by the presence of well-marked valves in the latter (Fig. 1g, h). The absence of the ‘forming fruit’ concept in current ontologies may not only constitute a gap and a semantic problem, but also may hinder the ability to define and classify RS in species with long reproductive cycles. Importantly, the lack of definition for this structure introduces a risk when combining disparate datasets for multiscale analysis if different researchers classify this stage differently (i.e., ‘fruit’ vs. ‘flower’).

The senesced flowers in eucalypts (Fig. 1f) may also constitute a semantic problem in the PPO. A flower is considered a ‘senesced flower’ (*sensu* PPO) when “all the petaloid floral organs have completed floral organ senescence”. In eucalypts, all the petaloid organs have dehisced at anthesis, which could indicate that the eucalypt flower transitions from ‘unopened flower’ to ‘senesced flower’. The previous statement is illogical to our understanding of eucalypt biology. Therefore, we suggest that the eucalypt flower be considered senesced when both the stamens and style have either abscised or senesced. We consider abscission alone insufficient, as style abscission does not occur in all species (e.g., *E. sieberi* and *E. leptophylla;* Ellis and Sedgley 1992; Bassett 1995); and stamens can be retained for a long time even when the fruit development is evident (Fig. S2 Online Resource 1).

### Mature fruits

For eucalypts, many terms have been used to describe the stage of the fruits; however, the most frequent terms are immature and mature capsules (e.g., Andersen 1989; Ashton 1975; Salter 2023). Dooley et al. (2010) defined the capsules as mature if the valves of the capsule opened after being dried, otherwise they were defined as immature. Koch (2003) defined mature fruits in *E. baxteri* as having seeds with a “dark brown seed coats and harder, white embryos”. Salter (2023) defined capsules as mature even if they did not have clear valve marks; Basset (2002), however, classified these as flowers. Consensus in the literature is that a capsule is both ripe and mature when it is fully grown, brown and woody, holds viable seeds, can dehisce, and is still attached to a living branch.

The concept of ripeness is not addressed in the literature on eucalypts, but it is an important concept in the PPO, and in the BBCH scale. Maturity and ripeness are, however, not synonymous. Gortner et al. (1967) explained that the concepts of maturation and ripening differ in that the former is initiated first while both finalize simultaneously. The maturation stage, *sensu* Gortner et al. (1967), starts after extensive cellular enlargement has occurred. This suggests that the term mature can be applied to a fruit when it is fully grown. Ripening, on the other hand, is a process that involves a mature fruit that undertakes a series of chemical and organoleptic changes. Although the concept of maturation does not exist in the PPO/PO, Gortner et al. (1967) and the PPO/PO agree on the concept of ripening. For eucalypts, we propose applying the term ‘immature capsule’ (Fig. 1g) to a fruit until it reaches its full size, and their valves are not developed. On the other hand, a capsule that is fully-grown with the valves well defined, but unopened, may be defined as a ‘mature capsule’. A ‘ripe fruit’ (Fig. 1i), however, is a capsule that has transitioned from green to brown without dehiscing. Based on these definitions, an ‘immature capsule’ is a synonym of ‘forming fruit’ and a ‘mature capsule’ a synonym of ‘ripening fruit’. When the ‘mature capsule’/‘ripening fruit’ is green, it is equivalent to a ‘unripe fruit’ (*sensu* PPO, see Fig. 1h), and when it is brown it should be considered a ‘ripe fruit’ (see Fig. 1i).

According to Gortner et al. (1967), the fruit finalizes its development when it has reached its maximum level of ripeness; a point that corresponds to the onset of the ‘fruit senescence’ (fruit aging that leads to decomposition). To date, neither PPO nor PO include the concept of fruit senescence. In eucalypts however, this stage is relevant, as it indicates the final stage of the reproductive cycle, from seed dehiscence to capsule fall, and accounts for fruits that might decay and never open. Semple and Koen (2020) referred to them as ‘over-mature’ or ‘dehisced capsule’. Here we suggest ‘senescing fruit’ (Fig. 1j) to maintain the PPO convention for fruits.

The proposed terminology for RS in eucalypts, as identified above, provides the foundation for the EPO. The EPO was created in a GitHub repository and can be accessed at the following url: https://github.com/ClaudHGE/EUCALYPTO-EPO.git. How these RS are related to each other, and the PPO is quantified in the next section.

## Quantitative relationships among reproductive structures: A case study of *Eucalyptus baxteri* s.l., subgenus *Eucalyptus*

### Methods

To test the applicability of the PPO and the EPO reproductive structure (RS) classes, we processed 239 herbarium sheets of *Eucalyptus baxteri* and *E. arenacea* (here after *Eucalyptus baxteri* s.l.) samples. The samples were collected from a range of sites spanning 136.78 to 149.38° E and 35.44 to 39.03° S, covering much of the longitudinal extent of the state of Victoria (Australia) and a section of the southeast of South Australia. Of those, we collected 134 samples from 2021 to 2023. Samples were collected in every season of the year. The remaining herbarium records were housed in the University of Melbourne Herbarium collection, which were collected from 1974 to 1987.

As different reproductive cohorts coexist in the same herbarium specimen, the data were collected independently per cohort. The umbel (cluster) was the main unit for data collection, which encompassed inflorescences as well as infructescences. Umbels exhibited different compositions of RS within a voucher. For example, some umbels had only open flowers, while others had both mature unopened flowers and open flowers. All the umbels in the record were counted and classified by cohort and umbel composition type.

All the umbels in each of the 239 samples were counted and classified by umbel composition type. For a maximum of five umbels per umbel composition type per cohort, all the RS were counted and classified by RS class, except for the ‘inflorescence bud’ and ‘bursting inflorescence bud’, where only the umbels were counted. With this information, a lower count value, average and standard deviation were calculated. The lower count was selected for further analysis to minimize the error of overestimation while still accounting for the abundance of structures at the umbel level, such as the bursting inflorescence buds and the inflorescence buds. This approach provided a factual measure that can be mapped to the whole plant (Brenskelle et al. 2019).

A Principal Component Analysis (PCA) was performed with the scaled abundance data to detect relationships and patterns between the RS classes of the EPO. PCA can provide insights into the congruence of proposed RS classes in the EPO with global frameworks. Furthermore, the dimensionality reduction provided by PCA could guide future studies based on phenological data collection in eucalypts. Two PCAs (PCA-PPOs) were performed to test the sensitivity of the PPO classification to the classification of ‘forming fruit’ as ‘senesced flower’ (PCA-PPOsw) or as ‘unripe fruit’ (PCA-PPOuf). In both analyses, ‘senescing fruit’ was combined with ‘ripe fruit’, and all the reproductive buds were grouped together as ‘unopened floral structure’. The PCAs were conducted using the packages tidyverse (v2.0.0; Wickham et al. 2019), factoextra (v1.0.7; Kassambara and Mundt 2020) and gridExtra (v2.3; Auguie 2017) of R (v4.2.2; R Core Team 2022) in RStudio (Posit team 2023). Varimax rotation was applied to the PCA results but did not change the internal structure of the identified components.

### Results

The PCA on the new eucalypt ontology (EPO) identified five components that explained 85% of the total variance in the composition and abundance of RS in the herbarium dataset, with the first three dimensions explaining 67%. For the PCAs using the PPO current classes, three components were identified in both analyses with 95% of the variance explained when the forming fruits were included as senesced flowers (PCA-PPOsw), and 98% when they were combined with unripe fruits (PCA-PPOuf).

**Fig. 2:**
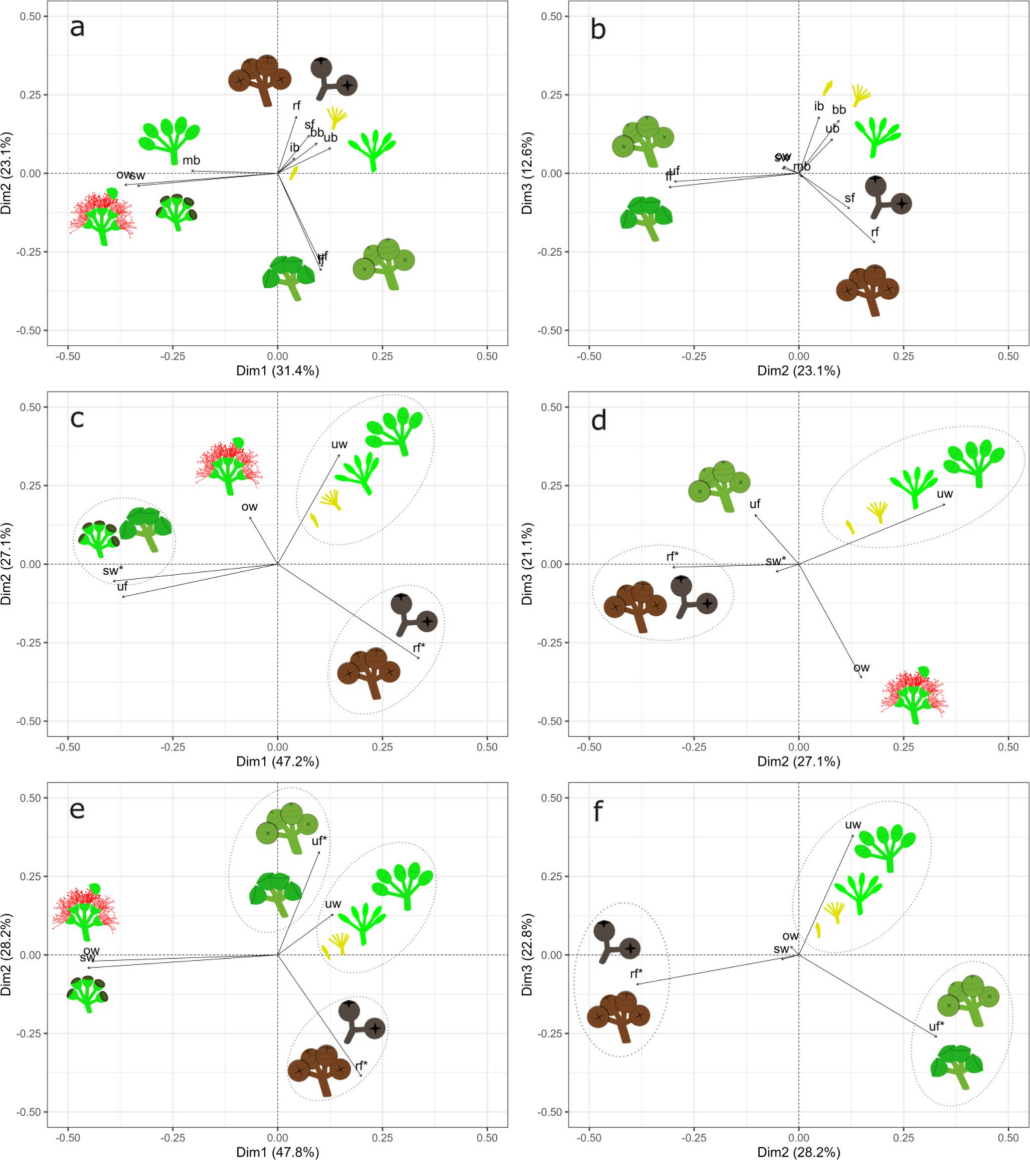
Principal Component Analyses (PCAs) plots of reproductive structure classes vs. normalized abundance per herbarium record. Top row (**a**, **b**) PCA-EPO: ten reproductive structure classes (RSC) created in the *Eucalyptus* Phenology Ontology (EPO). Middle row (**c**, **d**): PCA-PPOsw: four RSC existing in the Phenology Ontology Ontology (PPO) where the ‘forming fruit’ (ff) class was combined with ‘senesced flower’ (sw). Bottom row (**e**, **f**): PCA-PPOuf. RSC in the PPO classification where ‘ff’ was combined with ‘unripe fruit’ (uf). Ellipses in the PCA-PPOs indicate the RSC of EPO that were aggregated to conform the PPO classes. Asterisk (*) indicates a change in the meaning of the class. Abbreviations: ib: inflorescence bud, bb: bursting inflorescence bud, ub: intermediate bud, mb: mature bud, ow: open flower, sw: senesced flower, ff: forming fruit, uf: unripe fruit, rf: ripe fruit, sf: senescing fruit, and uw: unopened floral structure. Axis: Principal Components (Dim) and their respective percentage of variance explained. Plotted in R with the packages factoextra and gridExtra, graphics designed in inkscape (Inkscape Project 2020)

Principal component (PC) 1 explained 31.4% of the variance in the EPO analysis (Fig. 2a). The PC was characterized by a strong negative association of open flowers (ow) and senesced flowers (sw), followed by the mature buds (mb) (Fig. 2a; Table 1) in comparison to other structures. PC2 explained 23% of the variance and was negatively associated with forming fruits (ff) and unripe fruits (uf) and positively associated with ripe fruits (rf), senesced fruits (sf) and inflorescence, bursting inflorescence and intermediate buds (ib, bb and ub, respectively) or immature floral buds (Fig. 2a, b). PC3 explained 12.6% of the variance and was negatively associated with ripe and senescing fruits (rf and sf, respectively) and positively associated with immature floral buds (ib, bb and ub)(Fig. 2b). PC4 explained 10.7% and PC5 7.4% of the variance respectively. These latter two components highlighted the multi-cohort structure that can be present in eucalypts. PC4 was positively associated with inflorescence buds (ib) and ripe fruit (rf) and negatively associated with intermediate buds (ub) and senesced fruits (sf). PC5 was positively associated with senesced flowers (sw) and senesced fruits (sf) but negatively associated with mature buds (mb) (see Table 1).

**Table 1:** Total explained variance (in brackets) and loadings of three principal component analyses (PCAs) using the (1) *Eucalyptus* phenology ontology (EPO) terminology and the Plant Phenology Ontology (PPO). The forming fruits defined in the EPO were combined with the senesced flowers (2) or unripe fruits (PPO). Further amalgamations are expressed within the table. Asterisk (*) indicates a change in the meaning of the class. Variables are ordered by stage of development. Values in black-bolded are those with loading ≥|0.3|, grey-bolded values have a loading >|0.25|

1. Principal Component Analysis EPO. Loadings					
Reproductive Structure Classes	Comp.1 (31.4%)	Comp.2 (23.1%)	Comp.3 (12.6%)	Comp.4 (10.7%)	Comp.5 (7.4%)
Inflorescence bud (ib)	0.07	0.10	**0.48**	**0.57**	0.12
Bursting inflorescence bud (bb)	0.16	0.19	**0.46**	0.08	0.10
Intermediate bud (ub)	0.22	0.17	**0.29**	**-0.68**	-0.19
Mature bud (mb)	**-0.35**	0.01	-0.03	0.07	**-0.79**
Open flower (ow)	**-0.62**	-0.07	0.06	-0.10	0.09
Senesced flower (sw)	**-0.57**	-0.08	0.05	-0.12	**0.45**
Forming fruit (ff)	0.18	**-0.62**	-0.11	0.00	0.00
Unripe fruit (uf)	0.18	**-0.59**	-0.07	0.02	0.05
Ripe fruit (rf)	0.08	**0.36**	**-0.60**	**0.30**	0.01
Senescing fruit (sf)	0.13	0.24	**-0.29**	**-0.30**	**0.32**
2. **Principal Component Analysis PPOsw. Loadings**					
Reproductive Structure Classes	Comp.1 (47.2%)	Comp.2 (27.2%)	Comp.3 (21.1%)		
Unopened floral structure (uw = ib + bb + ub + mb)	0.23	**0.70**	**0.43**		
Open flower (ow = ow)	-0.10	**0.30**	**-0.82**		
Senesced flower (sw* = sw + ff)	**-0.60**	-0.11	-0.05		
Unripe fruit (uf = uf)	**-0.56**	-0.21	**0.36**		
Ripe fruit (rf* = rf + sf)	**0.51**	**-0.61**	-0.02		
3. **Principal Component Analysis PPOuf. Loadings**					
Reproductive Structure Classes	Comp.1 (47.8%)	Comp.2 (28.2%)	Comp.3 (22.8%)		
Unopened floral structure (uw = ib + bb + ub + mb)	0.19	0.25	**0.81**		
Open flower (ow = ow)	**-0.65**	-0.04	0.06		
Senesced flower (sw = sw)	**-0.66**	-0.08	-0.03		
Unripe fruit (uf* = uf + ff)	0.14	**0.63**	**-0.55**		
Ripe fruit (rf* = rf + sf)	**0.29**	**-0.74**	-0.20		

The PC1 of the PCA-PPOsw explained 47.2% of the total variance and was characterized by a strong negative association of the unripe fruits with the senesced flowers, and an important positive loading by the ripe fruits (Fig. 2c; Table 1). This component was mostly explained by post-anthesis structures. The second PC, which explained 27.2% of the variance, positively associated with unopened floral structures (uw) and negatively associated with ripe fruits (Fig. 2c, d). PC3 was negatively associated with open flowers (Fig. 2d).

For PCA-PPOuf, the first PC explained 47.8% of the variance and was strongly and negatively associated with open and senesced flowers (Fig. 2e; Table 1). PC2 explained 28.1% of the variance and had a negative association with ripe fruits and a positive association with unripe fruits (see Fig. 2e, f). The third PC explained 22.8% of the total variance and had a strong and positive association with unopened floral structures and a negative association with unripe fruits (Fig. 2f).

## Discussion

### Reproductive structures review

The inclusion of phenological traits in an ontology requires that reproductive structures (RS) are well defined, and their super and subclasses are clearly assigned. A review of eucalypt reproductive biology and phenology revealed the use of unstandardized terminology suggesting that RS in eucalypts are not defined consistently, which may present challenges for quantifying eucalypt phenology into the future. The PPO did not capture the complexity that characterizes eucalypt phenology. To address this, an extension to the PPO is proposed to account for the RS observed in eucalypts, the *Eucalyptus* Phenology Ontology (EPO). The proposed ontology includes more refined terms to more accurately describe the phenological traits of the eucalypts, while allowing them to be mapped into the PPO. The new ontology will be beneficial for facilitating the collation and harmonization of eucalypt phenological information within and outside of Australia as well as enabling the use of eucalypt data in global meta-phenological studies.

The new EPO may be useful for other taxa as well, especially those with long reproductive cycles. The entities proposed here are applicable to angiosperms in general as they go through a fruit formation stage (e.g., tomato: Quinet et al. 2019). Similarly, the EPO may be of use for other plant species that have a long floral structure development stage (e.g., Mao et al. 2017). Recording these different stages at finer resolution in these species could be of interest when annotating herbarium specimens. For example, phenologists could be interested in recording the ‘bursting inflorescence buds presence’ and ‘mature unopened flowers presence’ as they mark the beginning and the end of the ‘floral organ formation stage’ in addition to the fact that they might have been triggered by a climatic cue (Feng et al. 2021; Moncur 1992).

In eucalypts, having several classes for immature floral buds is critical for studying their phenological cycles and predicting future crops. As shown in Fig. S1 (Online Resource 1), eucalypt branches can exhibit several cohorts of RS, and more than one may contain floral buds. In species like *E. regnans*, where the ‘floral organ formation stage’ lasts more than two years and that new cohorts of floral buds emerge annually (Ashton 1975), having names for the different structures may aid cohort estimation and the prediction of the following years’ seed crop (Bassett 2011). In our analysis, this pattern was evident through the different relationships observed in PC3 and PC4. Immature floral bud classes were positively (PC3) or negatively (PC4) associated with each other indicating specimens can exhibit different patterns of immature bud development stages which may provide insights into the timing of phenophases and/or the impact of environmental factors on bud development. For example, the occurrence of abundant immature buds and no inflorescence buds may indicate that the younger cohort has finished the inflorescence bud stage, and no new floral structures are being initiated, or that the cohort of immature buds are in the second year of the ‘immature flower phase’ suggesting that inflorescence buds have not initiated yet or have failed to initiate. Conversely, a tree with abundant inflorescence buds and no immature buds would indicate that a tree is in the earliest part of the ‘immature flower phase’ and that inflorescence buds were not successfully formed in the previous year. To date, the reviewed ontologies do not offer this set of terminology.

In the case of the fruits, incorporating a class for senescing fruits may be convenient especially for dehiscent fruits (E. Denny, pers. comm., usanpn.org) as in some species they stay on the branches after dehiscence. The lack of clarity in the definition of “what a ripe fruit is” and “when the fruit is not ripe anymore” has brought serious difficulties in harmonization of datasets that the PPO does not address (E. Denny, pers. comm.). In response to this issue, we decided to follow Gortner et al. (1967) who provided physiological reasons for dichotomizing senescing from ripening fruit. In the case of eucalypts, we classified senescing fruits as those capsules that were dehisced and those that seemed to be decaying, which should be applicable for species with fruits that stay on the tree beyond ripeness.

### Associations between reproductive structures

#### Key phenophases in eucalypts

In the PCA-EPO, the immature buds (ib, bb and ub) were associated with the ripe and senescing fruits in the PC1 and PC2 (Fig. 2a quadrant I). The two groups diverge in PC3, indicating that in some samples, immature buds were present/abundant but ripe and senescent fruits were absent/scarce. PC4 highlighted that it was more likely for inflorescence buds and ripe fruits to co-occur and intermediate buds and senescent fruits to co-occur, indicating that samples can contain distinct phenological groups of cohorts. PC5 indicated that samples can have abundant mature buds but few, or an absence, of senesced flowers, and to a lesser extent senesced fruits. These relationships highlight the overlapping nature of the reproductive cycles on eucalypt branches, i.e., a new cohort of floral buds is developing while the older cohorts are in the ‘ripe fruit stage’. The PCA-EPO also captures the development within cohorts providing an indication of the progression through a phenophase. These associations were not observed in the PCA-PPOs.

One interesting association in the PCA-EPO, that was lost in the PCA-PPOs, is the positive correlation between the mature buds with open and senesced flowers (Fig. 2a). The generalization of unopened flowers in the PPO does not capture the key development steps leading up to flowering in eucalypts. The positive association in the PCA-EPO highlights that mature buds are an indicator of pending flowering. The strong negative association between mature buds and senesced flowers in PC5 supports this, as it indicates that a plant is in the early flowering stage. Samples with many mature buds and few or no senesced flowers are likely in the late flowering stage as evidenced by the mature bud– open flower relationships in PC1 and PC5.

In the PCA-EPO, ‘forming fruit’ strongly and positively aligned with ‘unripe fruit’ in the three dimensions evaluated (Fig. 2a, b). The two classes of fruits were negatively associated with the ‘open flower’ and ‘senesced flower’ in PC1. The two PCA-PPOs showed that the relations among the RS classes fluctuated according to the different ways in which the forming fruits were classified. These fluctuations were more relevant in ‘open flower’ vs. ‘senesced flower’ and the latter with ‘unripe fruit’. When ‘forming fruit’ was combined with ‘senesced flower’ (1) the correlation between ‘open flower’ and ‘senesced flower’ decreased dramatically, whereas (2) the correlation between ‘unripe fruit’ and ‘senesced flower’ changed from negligible to very strong. Given that the EPO has more refined definitions than the PPO, the PCA-EPO better grasps the variability and connections between the classes, even if the loadings are lower and the dimensions explain less of the overall variability. The connection between senesced flowers and unripe fruits might be considered artificial because of the amalgamation of classes. The PPO broader classification, however, may misrepresent the ‘fruit development stage’ in eucalypts.

The association of the RS classes in the PCA-EPO indicates that *Eucalyptus baxteri* s.l. has four broad phenophases:

1. *Immature flower phase*. Includes all the immature floral buds: inflorescence buds, bursting inflorescence buds and intermediate buds. It starts from meristem transition until the flower buds mature, exclusive.
2. *Mature flower phase*. Includes the mature flower organs: mature buds, open flowers and senescent flowers. It starts from the maturation of the floral organ to the end of the anthesis, inclusive.
3. *Non-ripe fruit phase*. Includes all the green fruits: forming fruits and unripe fruits. It starts with pollination and ends when the color of the fruit turns brown, exclusive.
4. *Post-ripeness phase*. Includes all the brown fruits: ripe fruits and senescing fruits. It starts from the coloration change to brown to fruit fall.

Here we used the word “phase” to prevent confusion with the ‘plant structure development stage’ of the PO or the growth stages of the BBCH. These phases do not align with the hierarchy defined in the PO and PPO ontologies nor do they coincide with the BBCH stages. The phases presented here were derived from the PCA, rather than the stages in the ontologies that are defined deductively based on the knowledge of plant reproductive biology. The main contrasts are presented as follows. First, the concept of mature and immature flower instead of the open and unopened flower of the PPO or, as in the BBCH: ‘inflorescence emergence’ (BBCH5) vs. ‘flowering stage’ (BBCH6). In the eucalypt ‘mature flower phase’, some unopened flowers are included as mature buds aligned with open and senesced flowers (Fig. 2a). Second, the concept of non-ripe fruits is represented in the ‘non-ripe fruit phase’, which includes all the fruits in the ‘fruit formation stage’ and some fruits in the ‘fruit ripening stage’. These stages are clearly differentiated in the PO and, also, in the BBCH as ‘development of the fruit’ (BBCH7) and ‘ripening or maturity of fruit and seed’ (BBCH8). Lastly, the ‘post-ripening phase’ coincides with the concept of ‘ripe fruit stage’ of the PPO, which does not acknowledge fruit senescence, nor does the BBCH. Our classification agrees with Gortner et al. (1967).

#### Implications for classifying phenological traits in eucalypts

The association between the RS classes in the PCA-EPO supported the importance of having narrower concepts for annotating traits in eucalypt species. For instance, the fact that mature buds correlated positively with open flowers and negatively with the rest of the floral buds highlighted that encapsulating all the floral buds in one structure class, as the PPO does, overgeneralizes the phenological responses of the eucalypts. This result indicates that the trait ‘mature flower bud present’ is a suitable indicator of a pending flowering event. Similarly, the lack of a term to define “the fruit that is forming”, as in the PO and PPO, implies that those fruits must be sorted either as a fruit or as a flower. The PCA-PPOs (Fig. 2c-f) demonstrated that the relationship between the classes fluctuated importantly according to how these structures, forming fruits, are classified, which indicates that the decision on their classification is not trivial. From a phenophase perspective, identifying these structures is important as the broader PPO classes would lead to mature buds being in the immature flower phase and, if merging forming fruits with senescent flowers, the senescent flowers would be merged with the non-ripe fruit phase (Fig. 2c), which could cause an overestimation of the fruit crop and a misidentification of the upcoming flowering event.

The tight association observed in PCA-EPO of forming and unripe fruits indicates that ‘forming fruit’ is more likely to represent flowering success than flowering. Not only are the forming fruits and senesced flowers different organs, but they also diverge ecologically, contrasting approaches by Basset (2002) and Brenskelle et al. (2019) in *Eucalyptus* spp. and *Prunus* spp., respectively. Despite their correlation, combining the forming fruits with unripe fruit is not plausible in order to maintain correspondence with the PO and PPO. The ‘unripe fruit’ is a subclass of ‘ripening fruit’, which is in the ‘fruit ripening stage’; whereas a ‘forming fruit’ is in ‘fruit formation stage’. A fruit can be either in the ‘fruit formation stage’ or in the ‘fruit ripening stage’, not both and not any. If the two are combined, they would need to be mapped as “developing fruit” which would include: ‘forming fruit’, ‘unripe fruit’ and ‘ripe fruit’. Such a class (‘developing fruit’) does not exist in the ontologies reviewed, so we have also created it in the EPO. To prevent overgeneralization, we recommend that distinctions are made between the ‘forming fruit’ and the ‘unripe fruit’ stages.

**Fig. 3:**
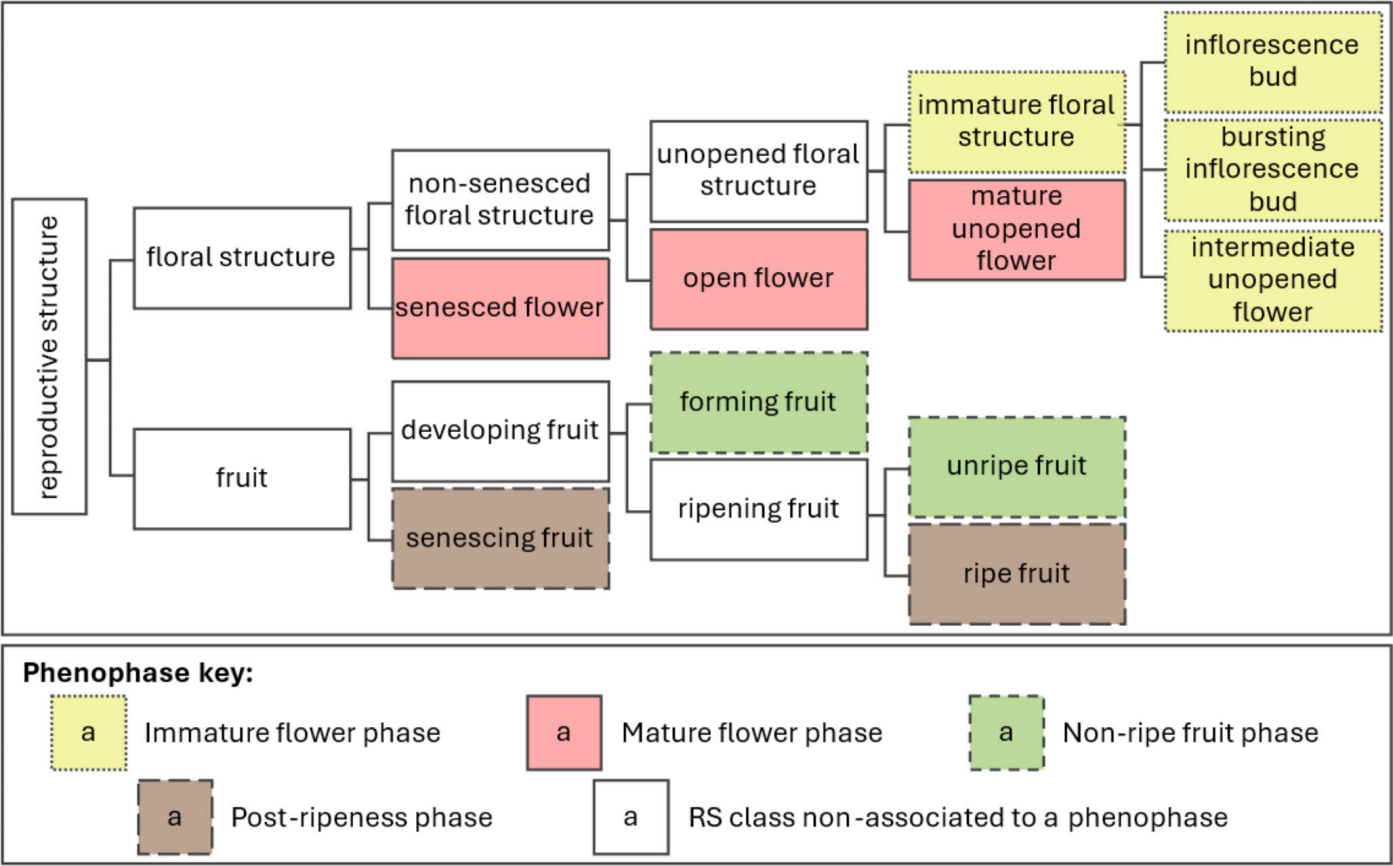
Hierarchical approach to defining the fruit and floral reproductive structures in eucalypts. The use of ‘floral structure’ can be changed to ‘flower’ or ‘inflorescence’ according to the characteristics of the plant. Two subclasses (‘flower’ and ‘inflorescence’) can be derived from every class of ‘floral structure’. The phenophases are indicated in the “Phenophase key” presented. The reproductive structures are ordered from top to bottom by level of development. RS: reproductive structure. For synonyms and taxa-specific terms see Fig. 1

The associations found in the PCA-EPO may serve as a guide to construct hierarchies that allow for generalization. Inflorescence buds, bursting inflorescence buds, and intermediate flower buds are likely to group together while diverging from mature flower buds. Although classifying them independently might serve various purposes, knowing how to group them allows the harmonization of datasets with different levels of detail. One of the advantages of ontologies is that they enable building diverse relationships that can be implemented according to the project needs. For instance, a suggested framework applicable to the subgenus *Eucalyptus* (Fig. 3) has segregated hierarchically the ‘unopened floral structure’ in a way that acknowledges the phenophase identified as the ‘immature flower phase’.

The phenophases identified with the PCA-EPO amplifies our understanding of the eucalypt reproductive ecology but, to guarantee data reusability, the associations found cannot be implemented for broadening the classification levels because they are not in agreement with the PO, PPO or BBCH scale. The sole phenophase that allows generalization is the ‘immature flower phase’ as all the respective RS can be classified as an ‘immature floral structure’ (Fig. 3). Such a class can be mapped as a subclass of ‘unopened floral structure’ (*sensu* PPO) or ‘reproductive bud’ (*sensu* PO). The ‘mature flower phase’ includes ‘mature unopened flower’, ‘open flower’ and ‘senesced flower’. These three classes cannot be combined as the PPO established that (1) ‘unopened flower’ shall be distinguished from ‘open flower’, (2) both ‘open flower’ and ‘unopened flower’ are a subclass of ‘non-senesced flower’, which shall be distinguished from ‘senesced flowers’ (Fig. 3). Therefore, if the three were combined they would map to the PPO as ‘flower’ or ‘floral structure’, resulting in an unacceptable loss of information. Similarly, that would be the case if ‘forming fruit’ and ‘unripe fruit’ are combined, which would map to ‘fruit’. Conversely, if ‘senescing fruit’ is combined with ‘ripe fruit’, they would map to ‘ripe fruit’ (*sensu* PPO) given that the PPO nor PO consider a ‘fruit senescing stage’ and those decaying fruits are included as ripe or excluded from the study (R. Walls, pers. comm., c-path.org). Generalizing up to the level of ‘unopened flower’ or ‘fruit’ might still be useful for species where the duration of the reproductive stages is short, like days or weeks, but in the case of some eucalypts, years of development may be reduced to a single state, and phenological patterns would be hard to detect. Furthermore, the fact that different cohorts overlap in a single individual makes phenological traits such as ‘unopened flowers present’ and ‘fruits present’ irrelevant, because under normal conditions they are always present.

In conclusion, if generalization is required, we stress the importance of recording independently, at minimum, ‘senesced flower’, ‘open flower’, ‘mature unopened flower’, ‘forming fruit’ and ‘unripe fruit’. If the ‘senesced flower’ or ‘senescing fruit’ are not relevant classes for the study, they can easily be ignored, which is preferred than combining them with another class. For eucalypts, we emphasize that distinctions should always be made between: (1) ‘forming fruit’, ‘unripe fruit’ and ‘ripe fruit’, and (2) ‘immature floral structures’, ‘mature unopened flower’ and ‘open flower’ to achieve not only compatibility with the global standards but also capturing the relevant phenological expressions in eucalypt trees. Lastly, implementing more refined classes will always be more beneficial as not only more diverse questions can be answered but harmonization through generalization to broader levels can be done while maintaining the finer resolution nature of the data. The EPO, as proposed, provides researchers with flexibility to work at the appropriate scale with consistent terminology when working within and across eucalypt species while enabling generalization to the PPO. This should facilitate the integration of species with complex phenology into global studies.

## Electronic Supplementary Material

Below is the link to the electronic supplementary material.

Supplementary Material 1
