# Supplementary material for: Definition of reproductive structures in Eucalyptus for phenological data collection
Source: Int J Biometeorol. 2024 Nov 14;69(11):2815–28. doi: 10.1007/s00484-024-02820-4 (PMC12540636; doi:10.1007/s00484-024-02820-4)
Supplement: Supplementary file 1 — Supplementary Material 1 [file 484_2024_2820_MOESM1_ESM.docx]

## **Supplementary information**

ONLINE RESOURCE 1


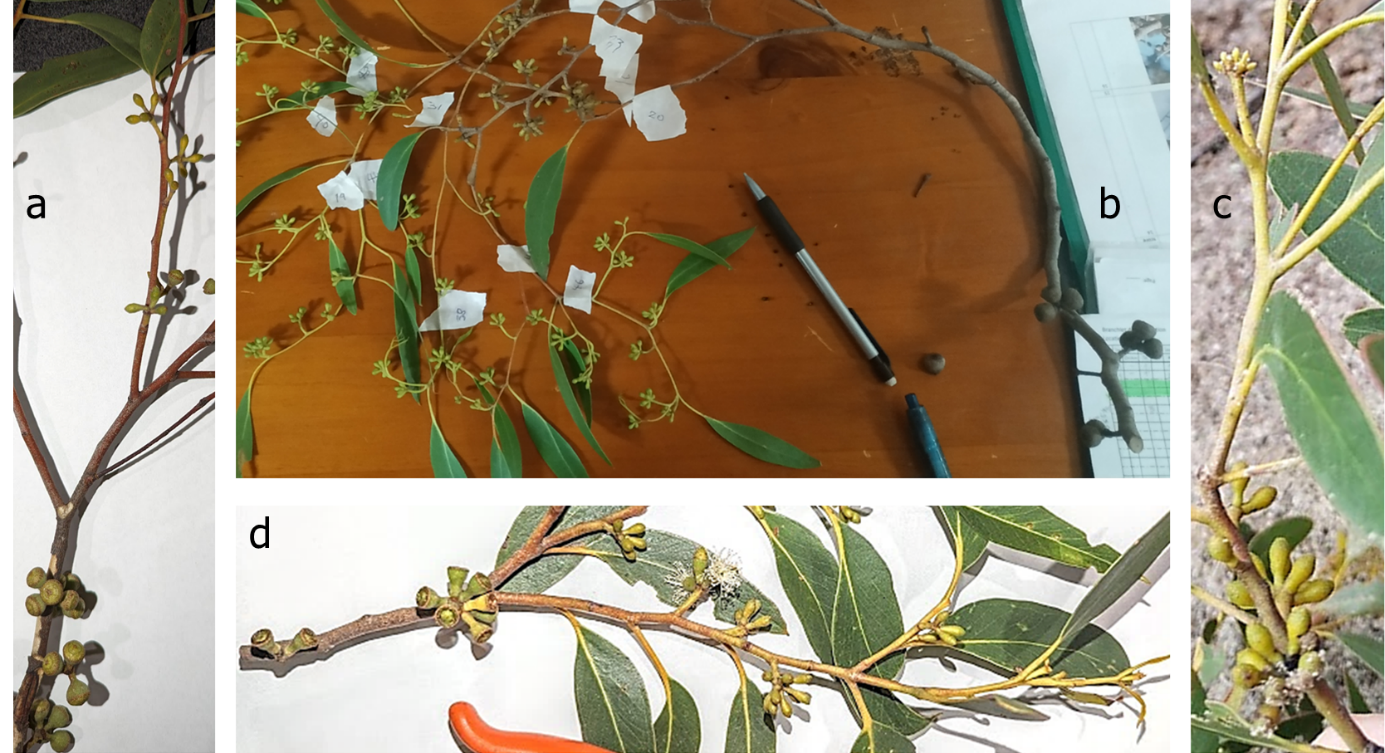


**Fig. S1** *Eucalypt baxteri* s.l. branchlets exhibiting various coexisting cohorts. (a) A younger cohort of flower buds and few forming fruits, and an older cohort of forming fruits. (b) Two cohorts of flower buds and one of ripe capsules. (c) One cohort of bursting inflorescence buds, and one of intermediate buds. (d) Three cohorts: one of inflorescence buds, another of intermediate buds and flowers, and one of forming fruits. Pictures: Claudia Helena Giraldo Escobar


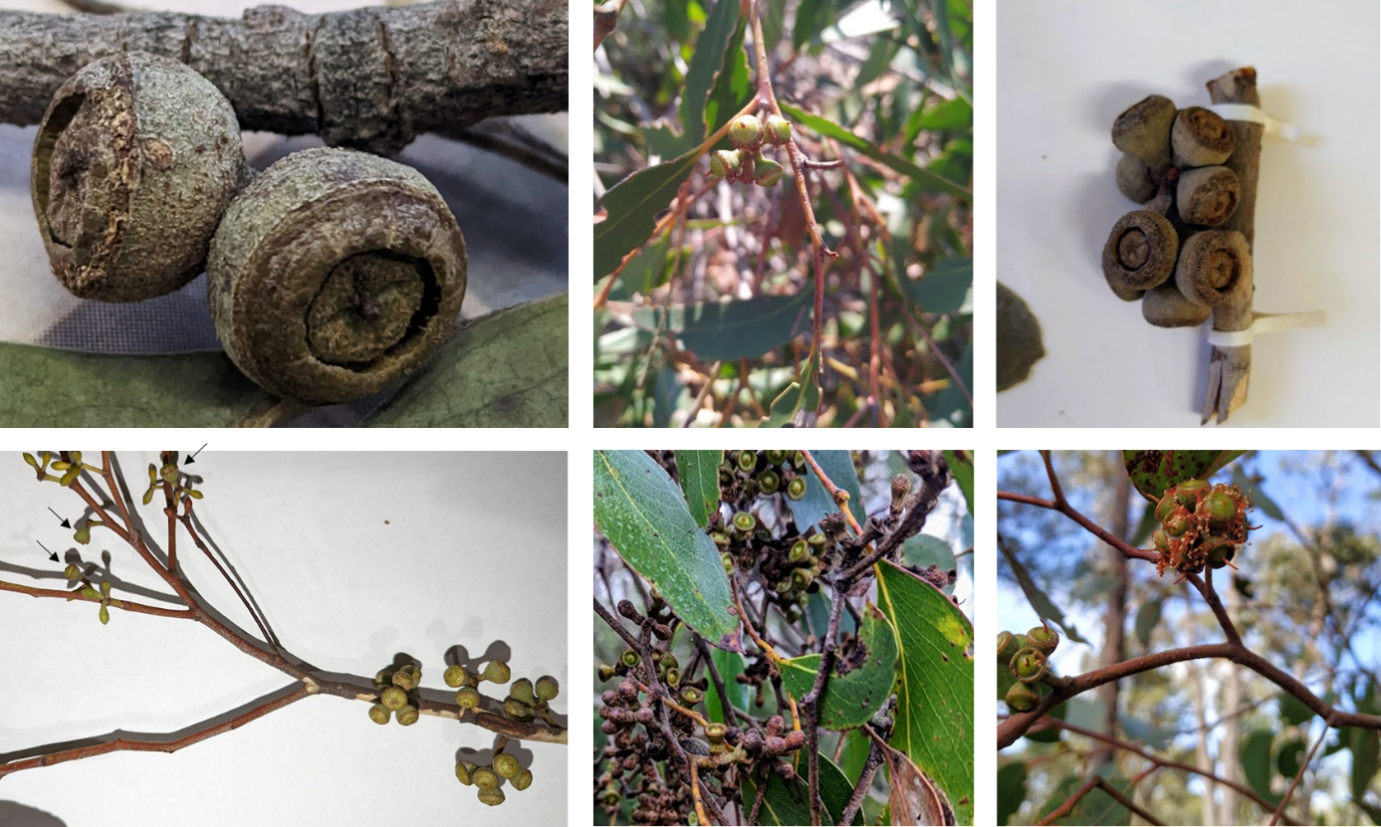


**Fig. S2** Forming fruits or immature capsules. Reproductive structures that showed signs of ovule swelling were classified as forming fruits, even if they had floral structures attached. Little arrows in the bottom left picture indicate forming fruits in the younger cohort. Pictures: Claudia Helena Giraldo Escobar
